# Barriers and facilitators to implementing polypharmacy management frameworks: a theory based qualitative exploration of key stakeholders

**DOI:** 10.1007/s11096-024-01844-5

**Published:** 2024-12-12

**Authors:** S. Al Bulushi, T. McIntosh, H. Talkhan, A. Grant, D. Stewart, M. Al Famy, S. Cunningham

**Affiliations:** 1 Ministry of Health, Muscat, Oman; 2 School of Pharmacy and Life Sciences, Applied Sciences and Public Health, Robert Gordon University, Garthdee Road, Aberdeen, AB10 7GJ UK; 3 Department of Clinical Pharmacy, College of Pharmacy, King Khalid University, Abha, Kingdom of Saudi Arabia; 4 School of Health, Robert Gordon University, Garthdee Road, Aberdeen, AB10 7GJ UK; 5 School of Pharmacy and Biomolecular Sciences, Royal College of Surgeons in Ireland, Dublin, Ireland

**Keywords:** Implementation science, Organisational change, Polypharmacy management, Strategic framework

## Abstract

**Background:**

Inappropriate polypharmacy arises through many factors including deficiencies in prescribing processes. Most research has focused on solutions at the clinician/patient levels with less at the organisational level.

**Aim:**

To explore key stakeholder identified barriers and facilitators to implementation of an organisational level polypharmacy management framework.

**Method:**

Qualitative data were collected within the Ministry of Health in Oman. Key stakeholders were purposively sampled encompassing senior representatives of pharmacy, medicine, and nursing directors; healthcare policymakers; patient safety leaders; and academic leaders. A semi-structured interview schedule was developed informed by a recent scoping review and underpinned by the Consolidated Framework for Implementation Research (CFIR). Interviews, which continued until data saturation, were audio-recorded, transcribed and analysed using the Framework Approach.

**Results:**

Thirteen key stakeholders were interviewed, with representation of each target group. Facilitators largely mapped to the CFIR domain of inner setting (i.e., aspects of stakeholder awareness, the electronic health system and national leadership), intervention characteristic (evidence gaps), characteristics of individuals (stakeholders and champions) and process (change strategy). Barriers also largely mapped to the inner setting (policy absence, communication and health professional practice) and outer setting (resource needs).

**Conclusion:**

This study has illuminated the facilitators and barriers to the implementation of an organisational level polypharmacy management framework. Further work is required to translate these themes into an actionable plan to implement the framework. Particular attention is required for aspects of the CFIR domain of inner setting (i.e., the internal context within which implementation occurs) as most barriers mapped to this domain.

## Impact statements

- Aspects of the inner setting (i.e., the internal context within which implementation occurs) are particularly important, acting as potential barriers to the implementation of a polypharmacy management framework.
- Focus should be placed on issues of national policy development, healthcare communication networks and health professional practice as part of strategic change relating to polypharmacy management.
- Facilitators such as national leadership and the data available in the electronic health systems should be highlighted in efforts to implement frameworks to overcome inappropriate polypharmacy.

## Introduction

The issue of preventable medication-related harm is widely acknowledged within global healthcare, as reiterated in a 2023 World Health Organization systematic review. One hundred studies involving 487,162 patients were included, with a pooled prevalence of preventable medication-related harm of 5% [1]. Of note, the highest prevalence was in geriatric care units (17, 95% Confidence Interval (CI) 4–35%, 9 studies). In an earlier systematic review, Hodkinson et al. also reported that the highest rates of preventable medication harm were in older patient care settings (11, 95% CI 7 to 15%) [2]. Such prevalence data are impacted by many issues including multimorbidity and polypharmacy [1]. Multimorbidity, defined as the presence of two or more chronic conditions in one person [3], is highly prevalent in older people who can be defined as individuals greater than 65 years of age (but definitions can vary) [4–6] and increases medication burden, often resulting in polypharmacy [7–9]. Traditionally defined as taking at least five medications on a regular basis [10], polypharmacy is regarded as one most complex prescribing problems [11, 12] with prevalence rates of 24–40% in Europe and the United States [13, 14] and 55–77% in the Middle East [15–17].

In 2017, the World Health Organization (WHO) launched ‘Medication without Harm’, with particular focus on 3 ‘key action areas’ of high-risk situations, transition of care, and polypharmacy [18]. The challenges articulated encompass strategies to mitigate the risks associated with polypharmacy, such as promoting appropriate prescribing practices, medication reconciliation, deprescribing, and enhancing patient education and engagement in medication management.

Polypharmacy is described as ‘appropriate’ (i.e., appropriate prescribing of multiple medications) and ‘inappropriate’ (prescribing of multiple medications which are either inappropriate or no longer indicated). Inappropriate polypharmacy can arise when the risks of using escalating numbers of medication outweigh the benefits [19]. There are a multitude of precipitating factors including system factors of a lack of clinical guidelines that support prescribing in older multimorbid patients [20] and deficiencies in the prescribing and deprescribing processes [21–24]. Common inappropriate polypharmacy medication-related problems include medication use without indication, duplication of therapy, drug interactions, adverse effects and poor adherence [25, 26]. Consequences of inappropriate polypharmacy are negative outcomes including adverse drug events, reduced functionality and significant health care costs [27–30]. Many have advocated for greater emphasis on reducing inappropriate polypharmacy in older people and ensuring rational prescribing based on the best available evidence, while taking account of individual patient factors and context [31–34]. The implementation of multidisciplinary guidelines that include interventions for healthcare practice has been the main strategy for change management to address inappropriate polypharmacy [35–37]. A Cochrane Review reported that of the included studies, the majority had interventions that were complex multi-faceted medication management focussed and directed at individual patients. The review concluded that there remained a lack of clarity on whether such interventions result in clinically significant improvements [33]. Medication reviews, when compared to standard care, have the potential to decrease hospital readmissions and emergency visits in adult patients who are hospitalised. However, their effect on mortality is minimal, and they may not have a significant impact on health-related quality of life [38]. Many guidelines and interventions focus on approaches related to rationalising medications for individual patients, often failing to consider the organisational context [10, 39]. Stewart et al. highlighted that only five European Union (EU) countries had guidelines on polypharmacy management in older individuals, and few existed elsewhere. None of the EU guidelines included change management strategies tailored to the organisational context [32]. Related work within the ‘Stimulating Innovative Management of Polypharmacy and Adherence in the Elderly’ (SIMPATHY) initiative summarised case studies from across Europe and highlighted the need to use theory-based implementation frameworks and organisational change management strategies for better implementation outcomes [39]. Al Bulushi et al. have published a scoping review, of 8 studies, characterising the literature on polypharmacy implementation frameworks, with focus on barriers and facilitators to organisational level implementation, identified only eight studies. Organisational level barriers included: poor organisational culture with a lack of sense of urgency and national plans, resource availability and communication issues including patient information and at transitions of care. Organisational facilitators included availability of government funding and regulatory environment promoting patient safety, a national emphasis on quality of care for older adults, co-ordinated national efforts and local evidence. The review concluded that in view of the limited literature there is a need for further research on implementation frameworks to foster effective organisational change [40].

### Aim

The aim was to explore key stakeholder identified barriers and facilitators to implementation of an organisational level polypharmacy management framework.

### Ethics approval

Ethical approval was obtained from Robert Gordon University (S293, November 2021) and Oman Ministry of Health Ethics Committee (MoH/CSR/21/25322, March 2022). Written, informed consent was received from all participants.

## Method

### Design

The study comprised qualitative, one-to-one, semi-structured interviews with key stakeholders.

### Setting

Data were collected from individuals in Oman where the Ministry of Health (MOH) oversees 268 health-care facilities across national, regional and local hospitals, and health centres providing primary, secondary and tertiary healthcare services [41].

### Participant sampling and recruitment

A purposive approach to sampling was employed targeting senior pharmacy, medical, and nursing directors; healthcare policymakers; patient safety leaders; and academic leaders responsible for developing, implementing, and evaluating healthcare policies and guidelines. Forty individuals were emailed to invite them to take part and from the responses initially 10 were selected for interview with the remainder held on record to meet the potential need for further interviews to reach data saturation. The sample size was based on the approach of Francis et al. [42] in interview based qualitative research using an initial sample size of 10 and a stopping criterion of 3. None of the invited participants refused to take part.

### Interview schedule development

**Fig. 1:**
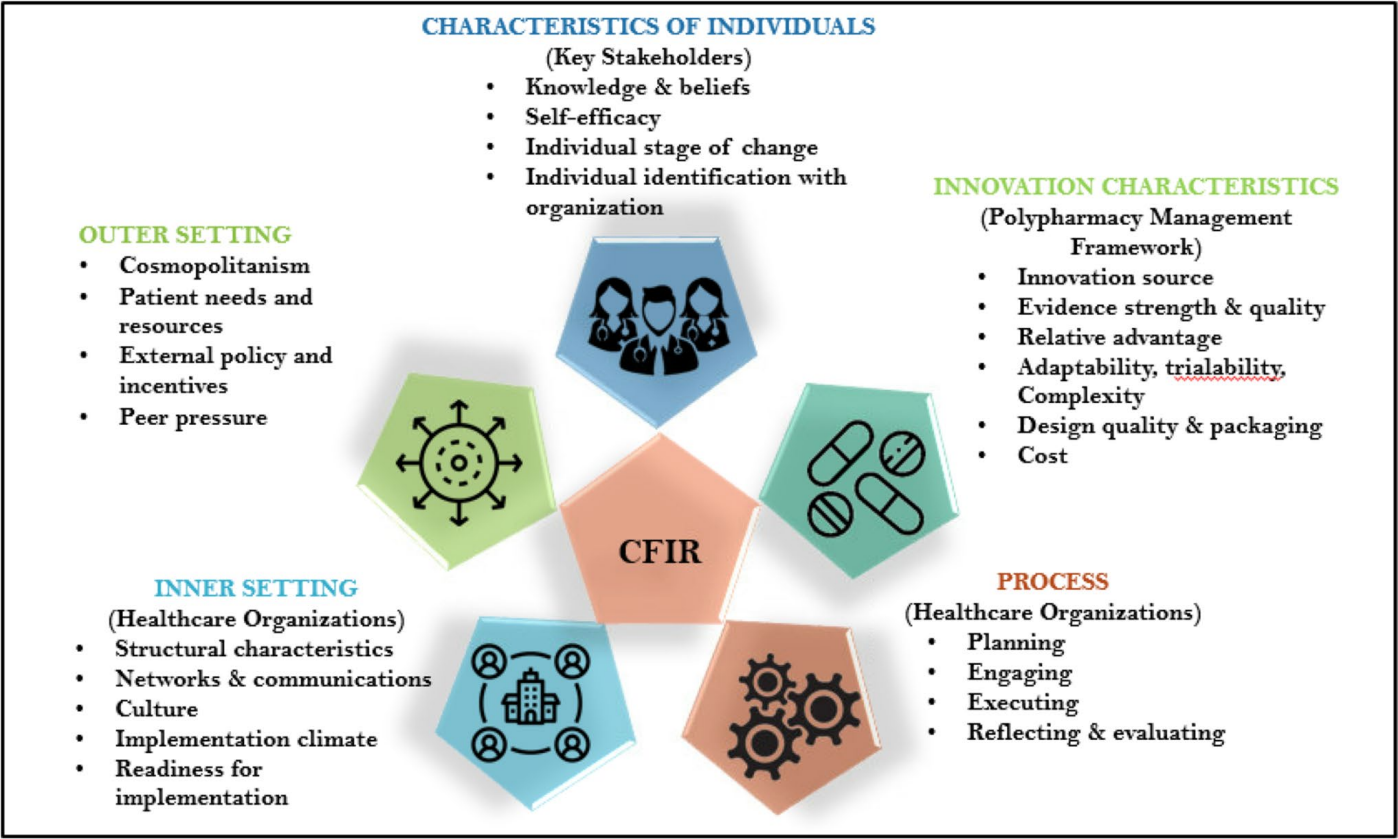
CFIR domains contextualised for organisational level polypharmacy management. Adapted from Damschroder et al. [43]

A semi-structured interview schedule was developed based on the findings of a recent scoping review [40]. The Consolidated Framework for Implementation Research (CFIR) provided the theoretical underpinning for the study. The questions were framed around CFIR, which has been widely used in the identification of implementation barriers and facilitators [43], and thus the likely determinants of successful implementation [44]. The original version of CFIR of 5 domains (Fig. 1) and 39 constructs was used in this study [43].

The interview questions were mapped to the CFIR domains, as shown in Table 1. The schedule was reviewed for credibility by members of the research team with extensive expertise in qualitative research, the use of CFIR and prescribing/polypharmacy research [45]. Pilot interviews were conducted with one pharmacy leader and one academic leader in Oman; pilot data were excluded from the dataset.

**Table 1:** Interview questions mapped to CFIR domains to the

Question	Probes	CFIR* domain
Polypharmacy is a focus of many healthcare organisations globally, What are your views about this?	With the third global patient safety challenge ‘Medication without harm’—WHO has selected polypharmacy as one of the priority areas to be addressed. What are your views of that? Other than WHO—what other policies or initiatives for polypharmacy management are you aware of? What are your views on the strength of evidence for strategies to improve polypharmacy? In Oman: what do think of the extent of the challenges around polypharmacy? what are the benefits of having a focus on polypharmacy compared to other priorities? what are you views on the need for national vision on implementing a polypharmacy management strategy?	Intervention characteristics Outer setting
What evidence or data on the challenge of polypharmacy is available to your knowledge in Oman?	For example, in relation to polypharmacy—would there be: data on drug related problems, prescribing data, emergency visits data, hospital admission data, other data? Based on the available data, can you comment on how—if at all—your organisation might prioritise a polypharmacy management strategy How could better data be used to help feedback to individuals and organisations to help improve practice in polypharmacy Do you feel it could be used to encourage or reward better practice?	Inner setting
What are your views on the need to change practice in relation to prescribing for people with polypharmacy?	How would changes fit with existing prescribing workflow and systems? How accepting do you think individuals would be of considering changes to prescribing practice for polypharmacy? Which aspects of the prescribing process could be changed or improved? What do you think the possible advantage of having a multidisciplinary team regarding polypharmacy management?	Inner setting
What type of teams need to be formed in order to implement polypharmacy management strategy?	Who do you think would be the key individuals to lead organisational change in polypharmacy management? Who else do you think could be part of the team to contribute to change? (Pharmacy, nursing…) Is there a vision for collaboration between physicians and pharmacists regarding polypharmacy management? (If Yes) what are the advantage of having a pharmacist in the team (If No) what do you think about it?	Inner setting
How do you think any strategy would be received by healthcare professionals that are responsible for implementing it?	What barriers would you think prevent or slow implementation of a polypharmacy management strategy? What are facilitators available at your organisation that enable the implementation of a polypharmacy management strategy? Do you think healthcare professionals need education around polypharmacy and at what level? Undergraduate, Continuing professional development etc.… Do you think there will be a local leadership support for the implementation of a strategy?	Characteristics of individuals process

^*^CFIR Consolidated Framework for Implementation research, *WHO* World Health Organization

### Data collection

Potential participants were invited by email to participate and given the option of the interview being conducted either face-to-face or online using Zoom Video Communications [46]. Interviews lasting between 30 to 45 min were conducted from March to November 2022. The interviews were conducted by SA, a doctoral student, and the Director of Pharmaceutical Care at MOH, who had received training in qualitative interviewing. In view of her position within the MOH SA was known by many of the research participants on a professional basis and they would have been aware of her commitments in relation to polypharmacy. The initial set of ten interviews took place in March 2023, followed by an additional three interviews in November of the same year. Interviews were audio-recorded, transcribed using Otter.ai [47] and verified for accuracy of transcription prior to analysis. Filed notes were taken immediately after each interview to capture notable thoughts and reflections on the process and initial insights into the data captured. Transcripts were not returned to the participants.

### Analysis

The 7 step Framework Analysis approach [48] was employed with data management facilitated through the use of QSR NVivo 20 software [49]. Data analysis was independently undertaken by SA and one other of SC, TM of HT, with a third researcher involved in cases of disagreement. Familiarisation took place by reading and rereading the transcripts [49, 50] followed by coding based on CFIR domains and constructs. A framework matrix was synthesised in NVivo to support the data charting of the data with the final step being data interpretation. Participants were not asked to provide feedback on the findings.

### Trustworthiness

Several steps were undertaken to promote research trustworthiness [51–53] in terms of: credibility (research familiarity with the culture in Oman, iterative questioning, expertise of the research team, use of CFIR; dependability (operational detail of data collection); confirmability (use of CFIR); and transferability (rich description of the research context). In addition, attention was paid to reflexivity throughout by the research team constantly considering how their subjectivity and context influence the research processes.

## Results

Data saturation was deemed to have occurred on completion of the thirteenth interview. The strategic positions and practice settings of the stakeholders are given in Table 2.

**Table 2:** Participants’ strategic positions and practice settings (N = 13)

Stakeholder category	Setting
Pharmacy practice leader	Primary, n = 1
	Tertiary, n = 2
Medical practice leader	Primary, n = 1
	Tertiary, n = 2
Nursing practice leader	Ministry of Health, n = 1
Health policy leader	Ministry of Health, n = 1
Patient safety leader	Ministry of Health, n = 2
Academic leader	College of Nursing, n = 1
	College of Pharmacy, n = 1
	College of Medicine, n = 1

### Themes

Themes of facilitators and barriers to implementing polypharmacy management frameworks mapped to the CFIR domains are given in Table 3.

**Table 3:** Themes of facilitators and barriers mapped to CFIR domains

Facilitators, Key themes [CFIR domain]	Barriers, Key themes [CFIR domain]
National and international evidence gaps on the challenges around polypharmacy [Intervention characteristics]	Resource needs at health care professional and patient level [Outer setting]
Awareness of stakeholders that polypharmacy management is a priority due to economic burden and patient safety [Inner setting]	Structural differences affecting communication and coordination of care across different health care settings [Inner setting]
Available features of electronic health system supporting polypharmacy management [Inner setting]	Healthcare professional practice factors affecting implementation [Inner setting]
National leadership support and stakeholders’ willingness to implement the polypharmacy management strategy [Inner setting]	Absence of a context specific policy or strategy for polypharmacy management [Inner setting]
Availability of capable and willing stakeholders and champions familiar with the concept of polypharmacy management and the need for change [Characteristics of individuals]	
Implementation of a strategy for change in polypharmacy management at organisational level by educating and engaging all stakeholders [Process]	

### Facilitators

1. National and international evidence gaps on the challenges around polypharmacy [CFIR domain, Intervention characteristics]

There was general recognition that polypharmacy is an issue globally, and specifically in Oman due to factors of chronic diseases in the aging population. Participants noted the desire to improve medication management and patient safety programmes based on international initiatives.

*“One of the activities of Ministry of Health is the first National Patient Safety Day…the WHO medication safety was launched for Middle East in Oman…This sort of activity indicates that the country is looking for better future for the management of medication.”* (Participant 4, Academic Leader Medicine)

2. Awareness of stakeholders that polypharmacy management is a priority due to economic burden and patient safety [CFIR domain, Inner setting]

Participants were acutely aware of the need to act at the organisational level of the healthcare system with anticipated benefits for the system as a whole and individual patients.

*“The policymakers here in Oman have to look into addressing the issue of polypharmacy as something that will benefit the whole healthcare system… The implications of the cost are an economic burden on the healthcare system.”* (Participant 6, Academic leader pharmacy)

*“The strategy will be more than welcomed because it will benefit everybody. It will benefit the patients in terms of reducing the comorbidities related to the polypharmacy. I think the enablers mainly here would be our national vision along with the different strategic plans in the Ministry of Health that are all geared towards improving patient experience, improving the quality of life of individuals and communities.”* (Participant 7, Nursing practice leader)

3. Available features of electronic health system supporting polypharmacy management [CFIR domain, Inner setting]

The availability of a high functioning and well-connected electronic health system to provide access to the information required to support the implementation of polypharmacy management in all settings was seen as an enabler.

*“We have electronic health records, and this is not available in every country in every hospital...We have a lot everywhere in Oman, electronic healthcare records, health records...So, I think you can do a lot through that and it will work.”* (Participant 3, Pharmacy Practice Leader)

*“Oman has a very connected network. If you talk about MOH* [Ministry of Health]*, we have this beautiful electronic health system…which brings us all together.”* (Participant 10, Medical practice leader 2)

4. National leadership support and stakeholders’ willingness to implement the polypharmacy management strategy [CFIR domain, Inner setting]

Participants expressed positive views regarding the support of leadership and their willingness to implement the polypharmacy strategy.

*“I think it’s possible to implement it* [polypharmacy management]* with the current resources that we have… when it comes to the leadership support.” *(Participant 11, Pharmacy practice leader 3)

5. Availability of capable and willing stakeholders and champions familiar with the concept of polypharmacy management and the need for change [CFIR domain, Characteristics of individuals]

Participants highlighted that there were individuals and professional groups well placed to tackle polypharmacy issues. There was specific discussion of the appropriateness of pharmacists having a particular role in polypharmacy management.

*“It is a medical necessity and we as healthcare providers need to appreciate all the concerns about polypharmacy, and we need to be prepared to deal with it. It’s absolutely necessary and we have to work on it.”* (Participant 13, Medical practice leader)

*“Pharmacists have been doing a lot, I think, through research… From their clinical areas they would maybe try to implement change in their respective areas.”* (Participant 3, Pharmacy practice leader)

6. Implementation of a strategy for change in polypharmacy management at organisational level by educating and engaging all stakeholders [CFIR domain, Process]

Participants acknowledged the significance of including all stakeholders in the implementation of a polypharmacy management strategy across all stages of health professional education, including undergraduate programmes. Participants also acknowledged the need to engage leaders and patients in the implementation of polypharmacy management.

*“Integration within health education programmes is needed. All the levels, even the bachelor’s level is very important because they’re the ones who are going to be working or training as students, and then practicing when they graduate.”* (Participant 7, Nursing practice leader)

*“There is a need to have more CPD* [continuing professional development] *related to this area* [polypharmacy], *covering all specialties not only GPs… all surgeons, all subspecialties in the system.”*

(Participant 9, Patient safety leader1)

*“Patient representation is key* [in the development]*. I think they need to be involved because patients are always put aside. They are always just receivers of good or bad care.”* (Participant 12, Patient safety leader 2)

### Barriers

1. Resource needs at health care professional and patient level [CFIR domain, outer setting]

Participants highlighted that there was recognition of the lack of resources for health professionals to allow them to deliver the care needed. Furthermore, patients are not actively involved in polypharmacy management, resulting in a loss of the important patient voice in management.

*“The overload of patient numbers visiting the organization…that’s another barrier. As a physician or a pharmacist, I would like to spend my time with my patient, go through everything, but at the same time, as a manager, you cannot ask me to see 100 patients and you expect me to give one hour for each.”* (Participant 5, Academic leader nursing)

*“One of the things that is usually left out is patient involvement. Having the patient on board is very important because these are the people who can tell us the difficulties that they’re facing and what they need, so involving them is something that we need to look at.”* (Participant 12, Patient safety leader 2)

2. Structural differences affect communication and coordination of care across different health care settings [CFIR domain, Inner setting]

Participants described the current organisational structure as being fragmented across the healthcare sectors. There appeared to be frustration that this could lead to a complex patient management system involving multiple professionals in different settings negatively impacting patient care, with high potential for error.

*“The challenge with us in Oman… we have different health care systems, government, private and also, we have sister government institutions…All of those electronic systems are not integrated together. You could have a patient going to one setup and then going to a different setup and getting similar medications or other different medications of different brands.”*

(Participant 7, Nursing practice leader)

3. Healthcare professional practice factors affecting implementation [CFIR domain, Inner setting]

In addition to the issue of fragmented systems, participants articulated aspects of healthcare professional practice which could impact polypharmacy management strategy implementation within the healthcare organisation. One key factor related to the absence of multidisciplinary working.

*“I think one of the challenges we have is the lack of multidisciplinary team management for the patients. I know very clearly that patients quite often are being exposed to fragmented care…so multidisciplinary management is one of the very weak areas that should be considered and looked at, and certain strategies should be or policies should be put into that… this will have a very direct effect on the polypharmacy management.”* (Participant 1, Health system expert)

4. Absence of a context specific policy or strategy for polypharmacy management [CFIR domain, Inner setting]

All participants highlighted the absence of local policies or strategies which specifically focused on polypharmacy management. The need for detailed performance indicators and outcome measure was articulated.

*“There is no protocol in Oman regarding the polypharmacy and how we manage the polypharmacy.”* (Participant 2, Pharmacy practice leader)

*“There should be a national policy and this policy should produce a strategy and a clear plan of action, with clear indicators or outcomes that should be put into to monitor the progress of the work.”* (Participant 1, Health system expert)

## Discussion

### Statement of key findings

Interviews with key stakeholders in Oman generated themes of facilitators and barriers to the implementation of an organisational level polypharmacy management framework. Facilitators largely mapped to the CFIR domain of inner setting (i.e., aspects of stakeholder awareness, the electronic health system and national leadership), intervention characteristic (evidence gaps), characteristics of individuals (stakeholders and champions) and process (change strategy). Barriers also largely mapped to the inner setting (policy absence, communication and health professional practice) and outer setting (resource needs).

### Strengths and limitations

One strength of this study was the use of CFIR to provide theoretical underpinning hence enhancing the likelihood of comprehensive coverage of implementation facilitators and barriers. This also adds to the evidence base of the application of theory in pharmacy related research [54]. Attention throughout was paid to the aspect of qualitative research trustworthiness (i.e., credibility, dependability, confirmability and transferability), as described earlier. The main limitation is that the data were collected from key stakeholders in Oman hence the data and conclusion may not be transferable to other settings and countries. Additionally, we recognise that the interview schedule questions (Table 1) could perhaps have been more expansive in the coverage of the CFIR domains and the predominant focus was in the ‘inner setting’ domains. However, we feel that valuable data have been generated despite this and that the themes identified do indeed show coverage of domains beyond the ‘inner setting’.

### Interpretation

Of note, the recent scoping review characterising the literature on polypharmacy implementation frameworks identified limited literature on facilitators or barriers to organisational level implementation [40].

The themes identified in this study as facilitators can be amplified to accelerate implementation. Of note, 3 themes mapped to the CFIR inner setting domain, i.e., the ‘setting in which the innovation is implemented’. The 3 themes of awareness of heightened priority, the electronic health system and capable individuals aligned with the constructs of structural characteristics, network and communication, culture, implementation climate and readiness for implementation [45].

Nguyen et al. identified clinician awareness of the risks of polypharmacy as facilitators for change [55] while Kardas et al. reported a lack of awareness of polypharmacy management guidelines in several European countries [56]. In a large European based consensus study of key stakeholders, Stewart et al. highlighted the need to raise awareness of issues relating to polypharmacy amongst health policy and amongst health professional leaders and to identify, share, disseminate, promote and support best practice [32].

The importance of a national electronic health system, digitalisation and the use of digital has also been highlighted by others [39, 57]. MacIntosh also found that countries that lacked polypharmacy initiatives were lacking data to promote a sense of urgency for change. The need for leadership support to facilitate implementation and behaviour change was also highlighted in a number of case studies conducted across European countries [39]. These findings align with the present study in terms of the facilitators related to CFIR domains of characteristics of individuals and process (i.e., activities and strategies used to implement the innovation). In the related field of deprescribing management, Scott et al., reported that beliefs about capabilities of health professionals to undertake their roles to be a facilitator [21].

Themes relating to the CFIR domain of outer setting (i.e., the setting in which the Inner Setting exists, e.g., hospital setting, health authority) did not emerge during interviews. This may be related to the types of individuals included in the study. Others have shown that aspects such as health authority structures and processes, including legislation are key to successful implementation [32, 39]

Those themes identified as barriers must be addressed and overcome to enable successful implementation. As with the facilitators, these largely mapped to the CFIR inner setting domain, specifically aspects of structural differences affecting communication and care, healthcare professional practice and policy absence. Others have reported similar findings in relation to polypharmacy management across Europe [39], medication management more generally [58], medication adherence [59] and medication reviews [60]. One benefit to using CFIR in this study is that the barriers can be used to inform the implementation strategies. Kirk et al., in a systematic review of the use of CFIR for implementation research reported the infrequent prospective use in strategy development [61].

Powell et al., developed the Expert Recommendations for Implementing Change (ERIC) list of strategies mapped to CFIR domains [62], which were later grouped into 9 clusters by Waltz et al. [63] and refined by Perry et al. [64]. In relation to this work the ERIC tool strategies help indicate potential ways to address the barriers identified in this study (Table 3). For example, several level 1 strategies (i.e strategies endorsed by at least 50% of respondents in the modified Delphi by Waltz et al. [63]) linked to the CFIR construct ‘Available Resources’ are relevant to the barrier identified in this study around ‘resources needs’ and include; conducting local needs assessment, involving patients/consumers and family members and obtaining and use patients/consumers and family feedback.

For ‘healthcare profession practice factors’ which links to the ‘Culture’ construct within CFIR the level 1 is; identify and prepare champions. For the barrier ‘Absence of a context specific policy or strategy for polypharmacy management’ (Table 3) which links to the CFIR ‘Available Resources’ construct the level 1 strategy is; access new funding.

Lastly, there are several level 2 strategies (i.e endorsed by 20–49.9% of respondents in the modified Delphi by Waltz et al. [63]) for the ‘Structural Characteristics’ construct and strategies include: assessing for readiness to identify barriers/facilitators and change physical structure and equipment.

### Further research

Further research should focus on translating these facilitators and barriers into an action plan for the implementation of an organisational level polypharmacy management framework. A consensus-based approach (e.g., Delphi technique) could be used with the same expert group stakeholders, with statements based on the CFIR derived themes of facilitators and approaches to overcoming the barriers.

## Conclusion

This study has illuminated the key facilitators and barriers to the implementation of an organisational level polypharmacy management framework in Oman. Further work is required to translate these themes into an actionable plan to implement the framework. Particular attention is required for aspects of the CFIR domain of inner setting (i.e., the internal context within which implementation occurs) as most barriers mapped to this domain.
